# Orbitofrontal control of conduct problems? Evidence from healthy adolescents processing negative facial affect

**DOI:** 10.1007/s00787-021-01770-1

**Published:** 2021-04-16

**Authors:** Boris William Böttinger, Sarah Baumeister, Sabina Millenet, Gareth J. Barker, Arun L. W. Bokde, Christian Büchel, Erin Burke Quinlan, Sylvane Desrivières, Herta Flor, Antoine Grigis, Hugh Garavan, Penny Gowland, Andreas Heinz, Bernd Ittermann, Jean-Luc Martinot, Marie-Laure Paillère Martinot, Eric Artiges, Dimitri Papadopoulos Orfanos, Tomáš Paus, Luise Poustka, Juliane H. Fröhner, Michael N. Smolka, Henrik Walter, Robert Whelan, Gunter Schumann, Tobias Banaschewski, Daniel Brandeis, Frauke Nees, IMAGEN Consortium

**Affiliations:** 1 Department of Child and Adolescent Psychiatry and Psychotherapy, Central Institute of Mental Health, Medical Faculty Mannheim, Heidelberg University, Square J5, 68159 Mannheim, Germany; 2 Department of Neuroimaging, Institute of Psychiatry, Psychology and Neuroscience, King’s College London, London, UK; 3 Discipline of Psychiatry, School of Medicine and Trinity College Institute of Neuroscience, Trinity College Dublin, Dublin, Ireland; 4 University Medical Centre Hamburg-Eppendorf, House W34, 3.OG, Martinistr. 52, 20246 Hamburg, Germany; 5 Medical Research Council, Social, Genetic and Developmental Psychiatry Centre, Institute of Psychiatry, Psychology and Neuroscience, King’s College London, London, UK; 6 Department of Cognitive and Clinical Neuroscience, Central Institute of Mental Health, Medical Faculty Mannheim, Heidelberg University, Square J5, Mannheim, Germany; 7 Department of Psychology, School of Social Sciences, University of Mannheim, 68131 Mannheim, Germany; 8 NeuroSpin, CEA, Université Paris-Saclay, 91191 Gif-sur-Yvette, France; 9 Departments of Psychiatry and Psychology, University of Vermont, Burlington, VT 05405 USA; 10 Sir Peter Mansfield Imaging Centre School of Physics and Astronomy, University of Nottingham, University Park, Nottingham, UK; 11 Department of Psychiatry and Psychotherapy, Charité, Universitätsmedizin Berlin, Campus Charité Mitte, Charitéplatz 1, Berlin, Germany; 12 Physikalisch-Technische Bundesanstalt (PTB), Abbestr. 2-12, Berlin, Germany; 13 Institut National de la Santé et de la Recherche Médicale, INSERM Unit 1000 “Neuroimaging and Psychiatry”, University Paris Sud, University Paris Descartes, Sorbonne Paris Cité, Paris, France; 14 Institut National de la Santé et de la Recherche Médicale, INSERM Unit 1000 “Neuroimaging and Psychiatry”, University Paris Sud, University Paris Descartes; Sorbonne Université, Paris, France; 15 Institut National de la Santé et de la Recherche Médicale, INSERM Unit 1000 “Neuroimaging and Psychiatry”, University Paris Sud, University Paris Descartes, Sorbonne Paris Cité, Orsay, France; 16 Bloorview Research Institute, Holland Bloorview Kids Rehabilitation Hospital and Departments of Psychology and Psychiatry, University of Toronto, Toronto, ON M6A 2E1 Canada; 17 Department of Child and Adolescent Psychiatry and Psychotherapy, University Medical Centre Göttingen, von-Siebold-Str. 5, 37075 Göttingen, Germany; 18 Department of Psychiatry and Neuroimaging Center, Technische Universität Dresden, Dresden, Germany; 19 School of Psychology and Global Brain Health Institute, Trinity College Dublin, Dublin, Ireland; 20 Department of Child and Adolescent Psychiatry and Psychotherapy, Psychiatric Hospital, University of Zurich, Zurich, Switzerland; 21 Zurich Center for Integrative Human Physiology, University of Zurich, Zurich, Switzerland; 22 Neuroscience Centre Zurich, University and ETH Zurich, Zurich, Switzerland; 23 Freie Universität Berlin, Berlin, Germany; 24 Humboldt-Universität Zu Berlin, Berlin, Germany; 25 Berlin Institute of Health, Berlin, Germany; 26 Physikalisch-Technische Bundesanstalt (PTB), Braunschweig, Germany; 27 Maison de Solenn, Paris, France; 28 AP-HP, Department of Child and Adolescent Psychiatry, Pitié-Salpêtrière Hospital, Paris, France; 29 Psychiatry Department 91G16, Orsay Hospital, Orsay, France

**Keywords:** Adolescence, Conduct problems, Subclinical, Affective processing, Orbitofrontal cortex, FMRI

## Abstract

Conduct problems (CP) in patients with disruptive behavior disorders have been linked to impaired prefrontal processing of negative facial affect compared to controls. However, it is unknown whether associations with prefrontal activity during affective face processing hold along the CP dimension in a healthy population sample, and how subcortical processing is affected. We measured functional brain responses during negative affective face processing in 1444 healthy adolescents [*M* = 14.39 years (SD = 0.40), 51.5% female] from the European IMAGEN multicenter study. To determine the effects of CP, we applied a two-step approach: (a) testing matched subgroups of low versus high CP, extending into the clinical range [*N* = 182 per group, *M* = 14.44 years, (SD = 0.41), 47.3% female] using analysis of variance, and (b) considering (non)linear effects along the CP dimension in the full sample and in the high CP group using multiple regression. We observed no significant cortical or subcortical effect of CP group on brain responses to negative facial affect. In the full sample, regression analyses revealed a significant linear increase of left orbitofrontal cortex (OFC) activity with increasing CP up to the clinical range. In the high CP group, a significant inverted u-shaped effect indicated that left OFC responses decreased again in individuals with high CP. Left OFC activity during negative affective processing which is increasing with CP and decreasing in the highest CP range may reflect on the importance of frontal control mechanisms that counteract the consequences of severe CP by facilitating higher social engagement and better evaluation of social content in adolescents.

**Supplementary Information:**

The online version contains supplementary material available at 10.1007/s00787-021-01770-1.

## Introduction

Conduct problems (CP) refer to a persistent pattern of antisocial behavior including aggressive, disobedient, rule-violating, deceptive, and destructive behaviors, which develop during childhood and adolescence and predict a variety of negative outcomes in life [1]. Understanding how CP from low to clinically relevant levels is associated with healthy adolescents might increase our understanding of the development of disruptive behavior disorders (DBD) such as conduct disorder (CD) or oppositional defiant disorder (ODD) [2]. Investigations of affective processing and its neurophysiological correlates may be fruitful in this context, as alterations in affective processing have often been reported in individuals with severe CP or DBD [3]. On a behavioral level, individuals with CP and DBD showed impaired recognition of fearful, sad, and happy facial expressions, while an impairment of the recognition of angry and disgusted faces was not consistently reported [4, 5].

Such affective processing has been linked, on a neural level, to the amygdala as one of the core regions [e.g., 6–9]. Previous studies in normative populations reported lower amygdala reactivity to affective stimuli was associated with increased antisocial behavior only in subgroups, e.g., when controlling for psychopathic traits [10, 11]. Importantly, prefrontal regions such as the ventromedial prefrontal cortex (vmPFC) and orbitofrontal cortex (OFC), including the anterior cingulate cortex (ACC), also play a critical part [9, 12–15]. In patients with CP and DBD, both adults and children, changes in prefrontal functioning were significantly associated with negative affective processing [16]. A meta-analysis by Yang and Raine [17] has shown a reduction of activation in frontal brain regions such as the OFC, ACC, and dorsolateral PFC (dlPFC) in individuals with antisocial behavior, which comprised patients with DBD. Rubia [18] also reports orbitofrontal-paralimbic dysfunction during affect regulation and Sterzer and colleagues [19] found a stronger reduction in the right dorsal ACC response during negative picture viewing in adolescents with CD compared to healthy control individuals. Moreover, adolescents diagnosed with DBD showed reduced vmPFC and medial OFC activity compared to healthy controls while viewing pictures of negative facial affect [20, 21]. However, reduced OFC responsivity during affective processing have not been consistently observed across studies in DBD. Increased functional OFC and ACC responsivity were also observed in children with early-onset childhood DBD for pain-related empathic processes [22] and some studies found no difference in prefrontal activity between DBD and control in boys [23, 24]. Some of the inconsistencies are likely to reflect the small sample sizes and design differences. Additionally, the impact of callous-unemotional (CU) traits on affective processing has been demonstrated in a number of studies, most prominently for the amygdala, showing hyperactivity in individuals with low CU traits and hypoactivity for individuals with elevated CU traits (for reviews, see [16, 25]). But prefrontal activation has also been shown to be affected by CU traits [26, 27].

Most studies to date have focused on the clinical diagnostic spectrum, and it remains unclear whether healthy adolescents with elevated CP, extending up to the clinical range but not meeting diagnostic criteria for DBD, also show altered subcortical or cortical prefrontal functioning during affective processing. This group may be of particular interest in terms of protective, control mechanisms to keep adolescents, despite high CP scores, from developing DBD. Importantly, a recently published paper by Spechler and colleagues [28] provided some evidence on an association between lower OFC volume and emotional and behavioral control in a non-clinical sample of dysregulated compared to control adolescents from the same study as reported here, however, the authors found no corresponding functional brain changes, and did not use a dimensional approach to investigate variations in conduct problems.

In this research, we focused on how high CP affects healthy adolescents´ functional brain responses to affective facial expressions in prefrontal regions (ACC and OFC) and the amygdala. In our categorical analysis, we compared closely matched groups with high and low CP levels, and hypothesized decreased prefrontal and amygdala activation in this non-clinical high CP sample. To address the often discrepant findings for healthy adolescents with CP and clinical groups with diagnosis of DBD, we also tested for linear and non-linear effects of the CP dimension across the full sample and in the high CP range on the ACC, OFC, and the amygdala [17, 29]. Testing within the high CP group could reveal subtle changes in brain responsivity that might be underestimated in categorical comparison and whole sample analyses, but still might be critical for those individuals who are at increased risk for DBD. Regarding CU traits, previous findings mostly refer to amygdala activity, and the goal of the present study was also to explore potential effects in the prefrontal region.

## Methods and materials

### Participants

**Fig. 1:**
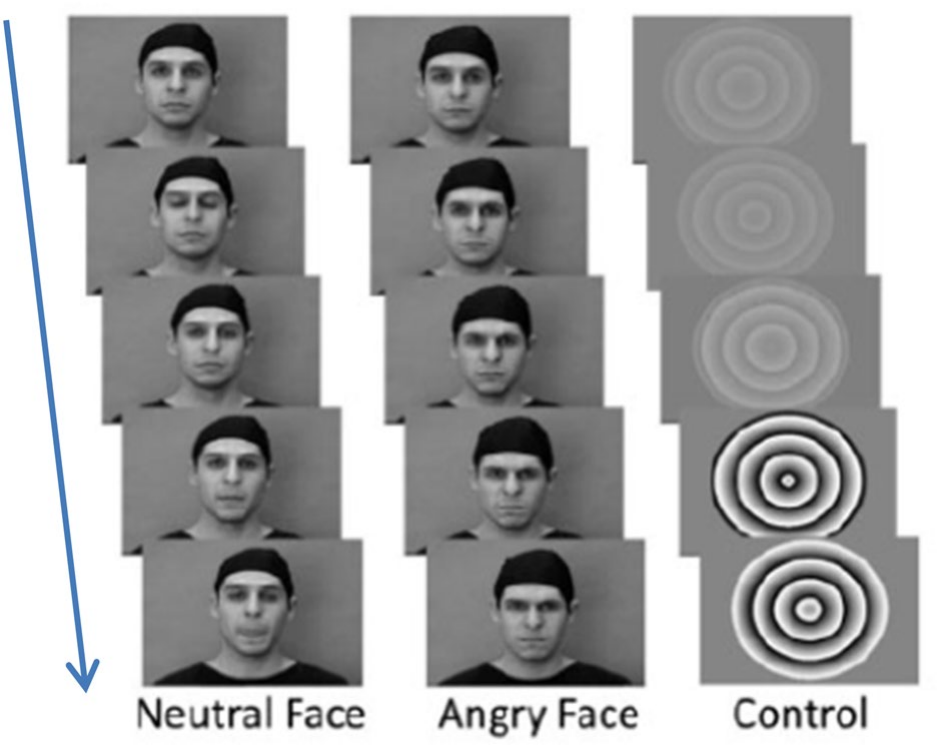
Faces task. Dynamic video clips of neutral faces, angry faces, and control stimuli conditions. Neutral faces either morphed into angry faces or displayed emotionally neutral movements

**Fig. 2:**
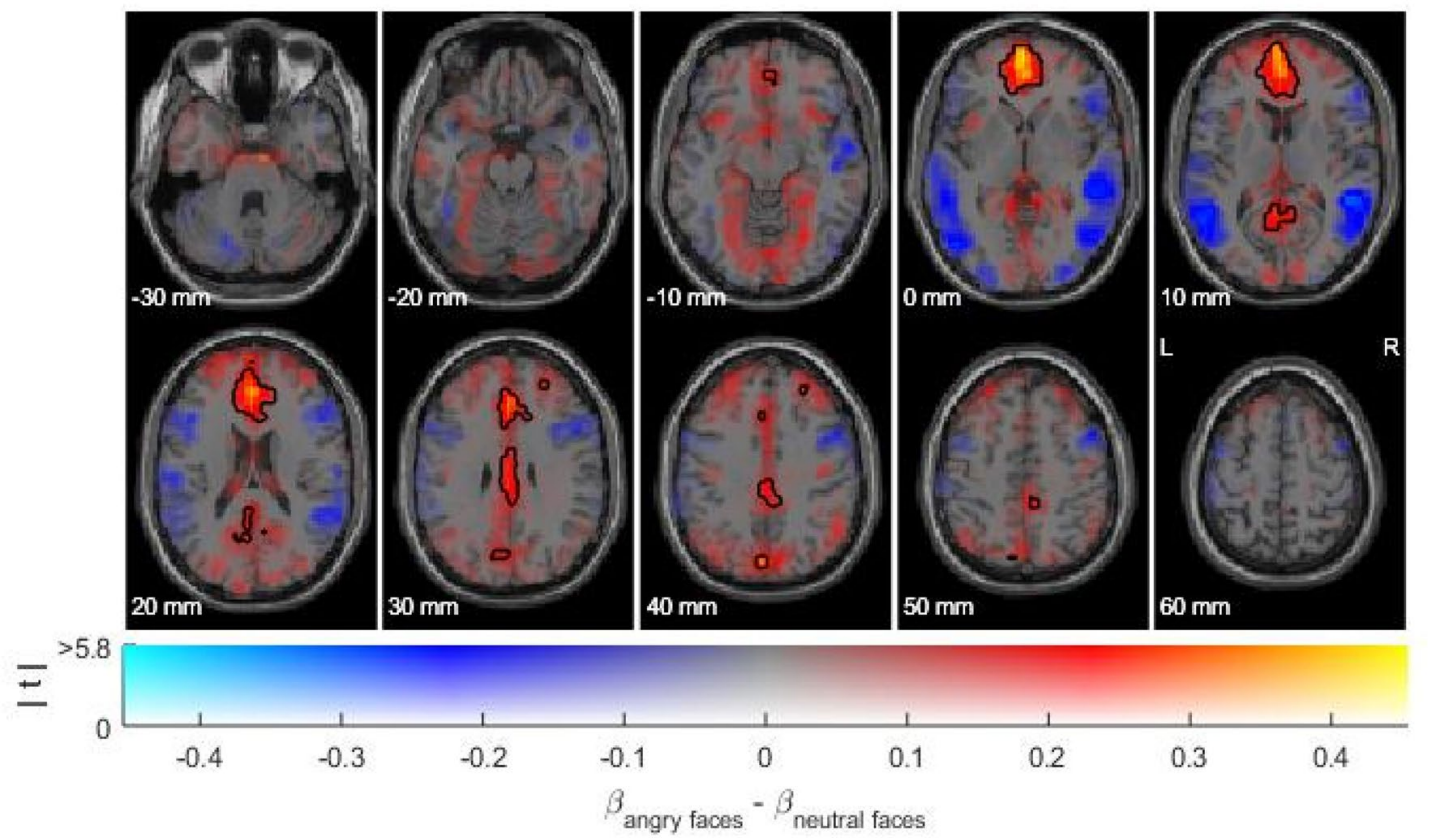
Task activation of the angry vs. neutral faces contrast (N_sub_ = 364). Simultaneous display of effect size (color-coded) and unthresholded *t*-statistics (opacity-coded). Black contours distinguish statistically significant and non-significant voxels at threshold *p* < 0.05 (FWE-corrected)

In the present study, we use data from the European multicenter study IMAGEN [30] where healthy adolescents were assessed at eight European sites across the United Kingdom (London, Nottingham), Ireland (Dublin), France (Paris), and Germany (Mannheim, Berlin, Hamburg, Dresden). For the purpose of the current study, we utilized the total available dataset (*N*_total_ = 1444) and selected adolescents who showed high levels of CP (*n* = 182) at the age of 14–15 years and individuals with low CP levels (*n* = 182), matched for sex, age, IQ, and pubertal development (*N*_sub_ = 364, *M* = 14.44 years (SD = 0.41), *n* = 174 female). For the definition of high versus low CP, we followed the clinical cutoff criteria of the Strengths and Difficulties Questionnaire’s subscale for CP (parent-rated version, cut off score = 4). Prosocial behavior, as an inverse proxy for CU traits, was higher in the control group, and used as a secondary covariate of interest in the analyses (see Table 1 below, and supplemental Fig. 1 for distribution of CP scores within the high and low CP groups) (Figs. 1, 2).

**Table 1:** Full sample and matched group characteristics

	Full sample	High CP	Low CP	Group comparison
	Mean (Std. Dev.)	Mean (Std. Dev.)	Mean (Std. Dev.)	*p* value
*N*	1444	182	182	
Matched variables				
PDS	7.52 (1.43)	7.37 (1.396)	7.49 (1.394)	0.404
IQ	108.14 (13.60)	106.70 (13.987)	106.69 (13.746)	0.994
Age	14.34 (0.95)	14.44 (0.412)	14.43 (0.408)	0.846
Sex (% female)	51.5%	47.3% (87)	47.3% (87)	0.295 (*χ*^2^ between) 0.459 (*χ*^2^ within)
SDQ domains				
Conduct problems	1.75 (1.57)	4.84 (1.042)	1.05 (0.763)	**
Prosocial behavior	7.91 (1.76)	6.54 (2.059)	8.23 (1.438)	**
Emotional problems	2.00 (2.02)	3.31 (2.462)	1.47 (1.569)	**
Hyperactivity	3.03 (2.28)	5.25 (2.334)	2.58 (1.958)	**
Peer problems	1.49 (1.59)	2.42 (2.071)	1.27 (1.422)	**
Total	8.27 (5.20)	15.82 (5.502)	6.38 (3.485)	**

*PDS* pubertal development scale, *SDQ* strengths and difficulties questionnaire, **p < 0.001

The study protocol was approved by the KCL (King’s College London) College Research Ethics Committee CREC/06/07-71 and by local ethics research committees at each site. Parents and adolescents gave written consent and verbal assent, respectively.

### Psychometric assessments

Conduct problems and prosocial behavior were assessed using the Strengths and Difficulties Questionnaire (SDQ), which is a brief behavioral screening questionnaire for individuals of 3–16 years and provides a dimensional measure of CP as well as emotional problems, peer problems, hyperkinetic symptoms, and prosocial behavior [31]*.* The status of pubertal development was assessed using the Pubertal Development Scale (PDS) [32] and IQ was estimated by averaging the sum scores of the Wechsler Intelligence Scale for Children (WISC-IV) subscales Matrix Reasoning (fluid IQ marker), and Vocabulary (crystalized IQ marker) [33].

### Experimental paradigm

Affective processing was assessed using a faces task from Grosbras and Paus [34]. In this task, participants were asked to passively view 18-s blocks comprising black-and-white video clips of faces and contracting or expanding concentric circles, which served as control stimuli. The face clips comprised five blocks of angry and neutral expressions each and were interleaved with nine blocks of the control stimuli (duration of each clip: 200–500 ms). In the angry face clips, faces with neutral expressions morphed into angry expressions, while in the neutral face clips, emotionally neutral expressions such as nose-twitching were presented. Each 18-s faces block contained 4–7 video clips.

### fMRI data acquisition

Magnetic resonance images were obtained on 3 T imaging systems (Siemens, Philips, GE, and Bruker). Four sites (using GE and Philips scanners) used an eight-channel coil, and four sites (using Siemens scanners) used a 12-channel coil. All sites used the same scanning protocol and image-acquisition techniques using a set of parameters compatible with all scanners were implemented to ensure a comparison of MRI [cf., 31]. To investigate and control overall quality characteristics of fMRI measurements between sites a fully-automated quality assurance using fMRI phantom measurements has been established [35].

A total of 160 volumes per subject were obtained, each containing 40 slices of 2.4 mm (1 mm gap), with a repetition time of 2.2 s and an echo time of 30 ms. Additionally, high-resolution T1-weighted three-dimensional structural images were acquired for anatomical localization and registration with the functional time series.

### fMRI data preprocessing and first level analysis

Data preprocessing and first level analysis were performed centrally at the Neurospin centre (at NeuroSpin-CEA, Gif-sur-Yvette, France) using the SPM8 software (http://www.fil.ion.ucl.ac.uk/spm/). Time series data were first corrected for slice-timing and then for movement (spatial realignment) relative to the first volume and non-linearly warped on the Montreal Neurological Institute (MNI) space, using a custom EPI template. Finally, images were smoothed with a Gaussian Kernel of 5 mm full-width half maximum. Individuals with anatomical abnormalities or excessive head movement (> 3 mm in at least one of the translations) did not pass quality control and were not included in the analyses (*n* = 20 of complete sample).

The single subject activation maps were computed within a general linear model (GLM) framework including 11 regressors modeling the experimental conditions (1 for each of the 5 angry and 5 neutral face video blocks, 1 concatenating all control stimuli) and convolved using SPM’s default hemodynamic response function. Estimated movement was added to the design matrix in the form of 18 additional columns (3 translations, 3 rotations, 3 quadratic and 3 cubic translations, 3 translations shifted 1 TR before, and 3 translations shifted 1 TR later). The estimated model parameters were then linearly combined in first level analyses to yield significance maps and contrast maps between the conditions. The contrast of interest for the present study was angry vs. neutral faces.

### fMRI data analyses of task and CP effects

To verify expected task activation in the subsample drawn for the purpose of the present study, contrast images were subjected to a one sample *t* test across all subjects. To additionally illustrate the whole pattern of neural activity from the angry vs. neutral faces contrast in the current subsample, the group-level contrast estimates were plotted using dual-coded design, which allows visualizing task-related threshold and sub-threshold activity [36, 37].

To determine effects of CP on brain responses during affective processing, we followed a two-step approach. Based on the previous findings from clinical versus control samples, we first tested for group effects of high versus low CP individuals using analysis of variance with the factor group and the angry faces vs. neutral faces contrasts. Second, in the light of contrasting findings in clinical and healthy samples, we tested for curvilinear relationships of CP and brain responses during affective processing in non-clinical populations, performing multiple regression analyses in the full sample and in the high CP group separately.

Effects were tested on the whole-brain level and the in regions of interests (ROIs) using SPM12 (http://www.fil.ion.ucl.ac.uk/spm/). ROI analyses were performed by extracting the individual mean contrast estimates within the selected ROIs (amygdala, OFC, and ACC) for the angry faces vs. neutral faces contrasts using masks from the Wake Forest University School of medicine (wfu) Human PickAtlas. Extracted contrast estimates were subsequently subjected to linear and non-linear regression analyses using SPSS software (Version 25, IBM Corp., Armonk, NY, USA).

Sex, age, PDS, IQ, site (dummy coded for whole-brain analysis), and prosocial behavior were included as control variables (covariates of no interest). As there is no direct measure of CU traits applied in the IMAGEN study, thus we approximated this important dimension using the prosocial SDQ subscale as an inverse proxy (i.e., low prosocial scores characterizing a lack of considerate, helpful, sharing behaviors as with high CU traits), as done in previous research, for example with younger groups [38]. Additionally, as the high and low CP groups were not matched for site, we tested for differences between sites within each group and for each covariate using analysis of variance (ANOVA). For whole-brain analyses family-wise error (FWE) correction for multiple testing was applied (critical α was set to 0.05) and for ROI analyses Bonferroni correction was applied, such that critical α was adjusted to 0.0083.

## Results

### Task effect

The angry faces vs. neutral faces contrast revealed activation in prefrontal areas comprising the medial frontal gyrus, anterior, and mid cingulate cortex, as well as the anterior insula and posterior regions including posterior cingulate cortex, cuneus, lingual and angular gyrus, and the fusiform gyrus. (For a complete list see supplemental Table A).

### Between group effects of high versus low CP

Whole brain analyses as well as ROI analyses of the amygdala, OFC, and ACC yielded no significant effect of CP group on brain responses during negative affective face processing. No significant effects of site on whole-brain activity could be detected in either group. Results of testing demographic and behavioral differences between sites within the high and low CP groups are reported in supplemental tables B1 and B2.

### Dimensional effects of CP on the amygdala

Whole brain and ROI analysis yielded no significant linear effects of CP on amygdala reactivity to negative affective stimuli in the full sample (supplemental tables C1 and C2).

### Dimensional effects of CP on OFC and ACC

In the full sample, we found a significant linear relationship between CP and left OFC activity (*R*^2^ = 0.010, *p = *0.048) where higher levels of CP were associated with higher left OFC activity (ß = 0.058, *p = *0.044, see Table 2). However, significance of this relationship did not survive control for multiple comparisons. When conducting the regression analysis without controlling for prosocial behavior, the effect of CP on left OFC activity was no longer significant (see supplemental tables F1 and F2). ROI analyses in the full sample yielded no significant dimensional effects of CP on the amygdala (supplemental tables C1 and C2), right OFC, and ACC (supplemental tables D1–D3).

**Table 2:** Full sample multiple regression on left OFC (angry > neutral) [model sig. *F*(7,1436) = 2.68 *p = *0.048]

Variables	*B*	Std. Error	Beta	*t*	Sig
(Constant)	− 0.326	0.267		− 1.222	0.222
**Conduct problem**	**0.010**	**0.005**	**0.058**	**2.015**	0**.044**
Prosocial behavior	0.010	0.004	0.066	2.311	0.021
Sex	0.016	0.014	0.029	1.087	0.277
Age	0.013	0.018	0.019	0.693	0.488
Pubertal development	0.009	0.005	0.048	1.746	0.081
IQ	0.000	0.001	− 0.021	− 0.774	0.439
site	0.002	0.003	0.018	0.669	0.503

Variable of interest is highlighted in bold

**Fig. 3:**
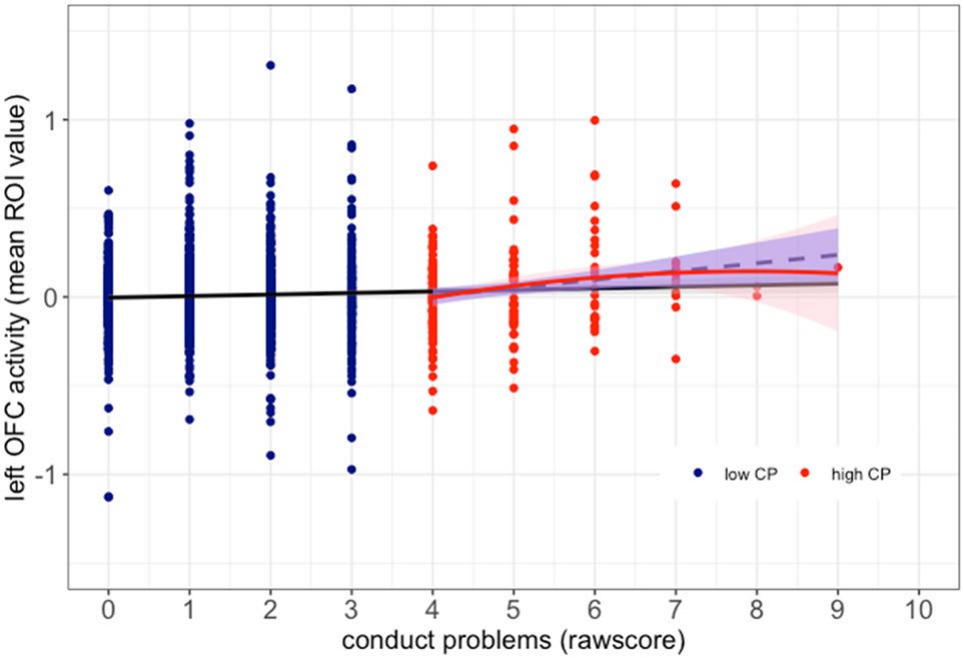
Dimensional effects of CP on left OFC activity. *Black line*: linear regression across the full sample. *Blue dashed line*: linear regression in the high CP group. *Red curve:* quadratic regression within the high CP group (inverted u-shaped). Shaded areas represent 95% confidence intervals

In the subgroup of participants with high CP, the linear relationship between CP and left OFC remained significant also when controlling for multiple comparisons (*R*^2^ = 0.110, ß1 = 0.253, *p* < 0.001, see supplemental table E1 and also supplemental table H1f or the matched sample (*R*^2^ = 0.047, ß1 = 0.21, *p* < 0.001). Additionally, we found a significant inverted quadratic association between CP and left OFC in this subgroup (*R*^2^ = 0.044, ß1 = 0.170, ß2 = − 0.011, *p = *0.018), with initially increasing activation with increasing CP and the slope turning negative again when CP were approximately around an SDQ score of 7 (see Fig. 3). After removal of three participants with exceptionally high CP scores (> 7) the inverted u-shaped relation in the group of adolescents with elevated CP remained significant (*R*^2^ = 0.048, ß1 = 0.062, ß2 = − 0.001, *p = *0.013). Such a quadratic association could not be observed in the full sample (*R*^2^ = 0.002, ß1 = − 0.002, ß2 = 0.002, *p = *0.313).

## Discussion

In the present study, we focused on the relationship between brain activation during negative affective processing and CP in an epidemiological adolescent sample. Dynamic stimuli of angry facial expressions, which have been previously shown to robustly elicit prefrontal activity comprising the ACC and OFC, were used to induce negative facial affect. Sex, pubertal development, the intelligence quotient (IQ), and site of data acquirement were used as covariates due to possible co-effects [39–42]. In dimensional analyses, we found linear and quadratic relationships between the level of CP and responses in the left OFC, but not ACC or amygdala, during the processing of negative facial expressions. In categorical comparison, there was no group difference between healthy participants with high compared to low levels of CP.

The non-significant group comparison between our healthy high and low CP groups might be seen in contrast to some studies in clinical samples reporting decreased OFC activity during negative affective face processing in adolescents diagnosed with DBD compared to healthy participants [20, 21], and observations of reduced orbitofrontal activation in relation to negative emotional stimuli in adult patients with impulsive aggression [43] or incarcerated adults with increased scores of psychopathy [22] compared to controls. However, similar to our findings, others have also failed to show prefrontal dysfunction in DBD compared to control participants [23, 24]. This might depend on the variance of CP levels and their linear composition within the healthy control groups and also due to the fact that our participants were still healthy adolescents (presupposing neural alterations only become significant in categorical group comparisons when symptoms are severe). Similar reasons might account for the non-significant group comparisons in the ACC. These null-findings might indicate that ACC functioning is a sensitive brain correlate in the clinical domain [e.g., 23], but no relevant brain correlate at higher, but still sub-clinical, CP levels in healthy individuals.

Further, we detected no significant group differences between high and low CP regarding amygdala responsivity. Although we have controlled for prosocial behavior as an inverse proxy for CU traits in the group comparisons, this result may reflect the previous finding that CU traits shape amygdala responsivity in different directions (for reviews, see Baker [25], Viding and McCrory [16]).

In dimensional analyses across all participants, activity in the left OFC linearly increased with increasing CP. Such linear associations have previously been observed for prefrontal volume and CP levels in healthy individuals, with elevated CP being related to increased volumes [29, 44]. Moreover, focusing on only those participants with elevated CP levels (i.e., SDQ score above 3) in our sample, revealed a quadratic association with an initially positive slope and a decrease in left OFC activity for the highest CP levels. This inverted u-shaped association remained significant when excluding subjects with exceptionally high CP levels. One might speculate that the positive association between OFC activity and elevated CP at least up to some high level reflects compensation processes, where increased OFC recruitment successfully counteracts less effective affective processing in the OFC through increased effort. Such reduced effectiveness of OFC activity can reflect educational deficits, increased peer-problems, or negative parenting styles [45–47] as well as higher perceived social uncertainty and irritability in social contexts [48, 49]. This potential compensation mechanism might particularly come into play in adolescence, a transition period where many social changes occur. For example, adolescents form more complex and hierarchical peer relationships and are more sensitive to acceptance and rejection by their peers than children [50, 51]. However, in adolescents with severely elevated CP levels, this compensatory function of the OFC is not present, which might increase the risk for future clinical diagnosis of DBD. The assumption of such a compensation mechanism might be tested by follow up observation of developmental trajectories in a longitudinal study design. As the IMAGEN study includes follow up assessments several years later, allowing the tracking of individual changes in SDQ scores and neural reactivity to affective stimuli, further analyses to test this specific hypothesis are intended by the authors.

Since the presence of elevated CP levels is not a sufficient criterion for the diagnosis of DBD, lacking information about frequency and persistence of symptoms, individuals with a confirmed clinical diagnosis of DBD likely represent subjects with more severe impairments. The inverted u-shaped association, if indeed extending into the clinical domain with increasingly reduced OFC activity, might suggest OFC hypoactivation is observed in group differences only when CP are severe enough. It further suggests that if CP levels are in a moderate range, OFC hyperactivation may be observed.

We observed no linear or non-linear associations between CP and ACC or amygdala activity. Although adult normative studies reported lowered amygdala responsivity with increasing antisocial, behavior when controlling for psychopathic traits [10, 11], our results suggest no association between amygdala activity and CP in adolescents, despite controlling for prosocial behavior as an inverse proxy for psychopathic or CU traits. Further, we found no dimensional relations between ACC activity and CP. Thus, these findings might also reflect a higher relevance of the OFC, compared to other regions, for the processing of anger stimuli (e.g., [47]).

## Limitations

While the present study provides important insight into the impact of CP on negative affective face processing, a number of limitations need to be considered. Most importantly, while the sample was large it did not specifically include patients with DBD and thus did not sufficiently cover the entire range of the SDQ CP subscale (scores from 0 to 10). Indeed, the number of subjects presenting with very high CP levels was low. Thus, the investigation of non-linear dimensional associations between CP and brain activity during affective processing needs to be urgently replicated in a sample extending well into the clinical range. Also, since several models were run, after correcting for multiple comparisons only the linear relation between the left OFC and CP in the high CP group remained significant. Further, De Brito and colleagues [29] have demonstrated an age effect on volumetric deviations in participants with elevated CP levels, thus arguing towards a lag in developmental maturation in participants with CP. In the present study, however, all participants were within a narrow age range. Therefore, despite controlling for age to account for subtle age differences, age effects could not be explicitly investigated here. Further, the quality control pipeline did not include quantification of signal loss at the individual level, which is particularly relevant for the OFC, thus potential signal loss was not controlled for.

The lack of group differences in the present study may be owed to the relatively small amount of individuals displaying very high levels of CP, who also likely fulfill diagnostic criteria of DBD.

Further, as we used prosocial behavior as an inverse proxy to CU traits in adolescents, assessing also other facets of behavior, possible effects of psychopathic or CU traits might be diluted in this study. Moreover, in our sample we detected sex differences regarding prosocial behavior, emotional problems, and peer problems as well as in IQ and PDS. However, also to account for previous findings of sex differences in internalizing and externalizing behavior [52–54] and the developmental differences in intelligence performance in boys versus girls [55], we further controlled for sex, IQ and PDS when possible.

As the faces fMRI task did not require behavioral or rating responses of the subject, we had no control on whether our participants were attentive to the stimuli during the presentation. However, as they were all healthy we have no reason to assume major shifts in attention or movement due to hyperactivity. Finally, it should be noted that the fMRI data were preprocessed with a somewhat outdated software pipeline relying on SPM 8, consistent with previous IMAGEN publications. We did not expect major differences due to an updated software pipeline, and thus decided to keep the original preprocessing also for the current analyses for compatibility reasons.

## Conclusions

In this study, we observed an increase with a non-linear, u-shaped component in left OFC response to negative affective face processing with increasing CP levels in a healthy adolescent sample. This pattern in the sub-clinical range stands in contrast to most findings known from clinical groups with DBD, where a decreased response has often been reported [19–21]. However, we also observed a non-linear, inverted u-shaped effect of CP in those individuals with elevated CP levels. This indicates that for those individuals with the highest CP levels, OFC responsivity decreases again. This activity pattern might suggest a compensatory mechanism where increased OFC activity might counteract the consequences of severe CP by facilitating higher social engagement and better evaluation of social content, which might result in better socially adapted behavior. Such compensation might fail when CP levels are very high and far in the clinical range, resulting in less adapted social behavior. As the present sample lacks sufficient coverage of very high CP levels, future studies including clinical samples of adolescents with diagnosis of DBD and adequately sized groups of healthy adolescents with exceptionally high CP are urgently needed to validate the suggested frontal compensatory mechanism.

## Supplementary Information

Below is the link to the electronic supplementary material.

Supplementary file1 (DOCX 139 KB)

## References

[1] Odgers CL, Caspi A, Broadbent JM, Dickson N, Hancox RJ, Harrington H (2007). Prediction of differential adult health burden by conduct problem subtypes in males. Arch Gen Psychiatry.

[2] Loeber R, Burke JD, Lahey BB, Winters A, Zera M (2000). Oppositional defiant and conduct disorder: a review of the past 10 years, part I. J Am Acad Child Adolesc Psychiatry.

[3] Viding E, Sebastian CL, Dadds MR, Lockwood PL, Cecil CA, De Brito SA (2012). Amygdala response to preattentive masked fear in children with conduct problems: the role of callous-unemotional traits. Am J Psychiatry.

[4] Fairchild G, van Goozen SH, Calder AJ, Goodyer IM (2013). Research review: evaluating and reformulating the developmental taxonomic theory of antisocial behaviour. J Child Psychol Psychiatry.

[5] Blair RJR, Veroude K, Buitelaar JK (2018). Neuro-cognitive system dysfunction and symptom sets: a review of fMRI studies in youth with conduct problems. Neurosci Biobehav Rev.

[6] Phelps EA, LeDoux JE (2005). Contributions of the amygdala to emotion processing: from animal models to human behavior. Neuron.

[7] Lindquist KA, Satpute AB, Wager TD, Weber J, Barrett LF (2016). The brain basis of positive and negative affect: evidence from a meta-analysis of the human neuroimaging literature. Cerebral Cortex (New York, NY: 1991).

[8] Müller VI, Höhner Y, Eickhoff SB (2018). Influence of task instructions and stimuli on the neural network of face processing: an ALE meta-analysis. Cortex.

[9] García-García I, Kube J, Gaebler M, Horstmann A, Villringer A, Neumann J (2016). Neural processing of negative emotional stimuli and the influence of age, sex and task-related characteristics. Neurosci Biobehav Rev.

[10] Hyde LW, Byrd AL, Votruba-Drzal E, Hariri AR, Manuck SB (2014). Amygdala reactivity and negative emotionality: divergent correlates of antisocial personality and psychopathy traits in a community sample. J Abnorm Psychol.

[11] Hyde LW, Shaw DS, Murray L, Gard A, Hariri AR, Forbes EE (2016). Dissecting the role of amygdala reactivity in antisocial behavior in a sample of young, low-income, urban men. Clin Psychol Sci.

[12] Davidson RJ, Putnam KM, Larson CL (2000). Dysfunction in the neural circuitry of emotion regulation–a possible prelude to violence. Science (New York, NY).

[13] Davidson RJ, Jackson DC, Kalin NH (2000). Emotion, plasticity, context, and regulation: perspectives from affective neuroscience. Psychol Bull.

[14] Yang M, Tsai SJ, Li CR (2020). Concurrent amygdalar and ventromedial prefrontal cortical responses during emotion processing: a meta-analysis of the effects of valence of emotion and passive exposure versus active regulation. Brain Struct Funct.

[15] Riedel MC, Yanes JA, Ray KL, Eickhoff SB, Fox PT, Sutherland MT (2018). Dissociable meta-analytic brain networks contribute to coordinated emotional processing. Hum Brain Mapp.

[16] Viding E, McCrory EJ (2018). Understanding the development of psychopathy: progress and challenges. Psychol Med.

[17] Yang Y, Raine A (2009). Prefrontal structural and functional brain imaging findings in antisocial, violent, and psychopathic individuals: a meta-analysis. Psychiatry Res.

[18] Rubia K (2011). “Cool” inferior frontostriatal dysfunction in attention-deficit/hyperactivity disorder versus “hot” ventromedial orbitofrontal-limbic dysfunction in conduct disorder: a review. Biol Psychiat.

[19] Sterzer P, Stadler C, Krebs A, Kleinschmidt A, Poustka F (2005). Abnormal neural responses to emotional visual stimuli in adolescents with conduct disorder. Biol Psychiat.

[20] Passamonti L, Fairchild G, Goodyer IM, Hurford G, Hagan CC, Rowe JB (2010). Neural abnormalities in early-onset and adolescence-onset conduct disorder. Arch Gen Psychiatry.

[21] Fairchild G, Hagan CC, Passamonti L, Walsh ND, Goodyer IM, Calder AJ (2014). Atypical neural responses during face processing in female adolescents with conduct disorder. J Am Acad Child Adolesc Psychiatry.

[22] Decety J, Skelly L, Yoder KJ, Kiehl KA (2014). Neural processing of dynamic emotional facial expressions in psychopaths. Soc Neurosci.

[23] Herpertz SC, Huebner T, Marx I, Vloet TD, Fink GR, Stoecker T (2008). Emotional processing in male adolescents with childhood-onset conduct disorder. J Child Psychol Psychiatry.

[24] Jones AP, Laurens KR, Herba CM, Barker GJ, Viding E (2009). Amygdala hypoactivity to fearful faces in boys with conduct problems and callous-unemotional traits. Am J Psychiatry.

[25] Baker RH, Clanton RL, Rogers JC, De Brito SA (2015). Neuroimaging findings in disruptive behavior disorders. CNS Spectr.

[26] Sebastian C, McCrory E, Dadds M, Cecil C, Lockwood P, Hyde Z (2014). Neural responses to fearful eyes in children with conduct problems and varying levels of callous–unemotional traits. Psychol Med.

[27] Hwang S, Nolan ZT, White SF, Williams WC, Sinclair S, Blair RJ (2016). Dual neurocircuitry dysfunctions in disruptive behavior disorders: emotional responding and response inhibition. Psychol Med.

[28] Spechler PA, Chaarani B, Orr C, Mackey S, Higgins ST, Banaschewski T, et al. (2019) Neuroimaging Evidence for Right Orbitofrontal Cortex Differences in Adolescents With Emotional and Behavioral Dysregulation. J Am Acad Child Adolescent Psychiatry 10.1016/j.jaac.2019.01.021 31004740

[29] De Brito SA, Mechelli A, Wilke M, Laurens KR, Jones AP, Barker GJ (2009). Size matters: increased grey matter in boys with conduct problems and callous-unemotional traits. Brain: J Neurol.

[30] Schumann G, Loth E, Banaschewski T, Barbot A, Barker G, Buchel C (2010). The IMAGEN study: reinforcement-related behaviour in normal brain function and psychopathology. Mol Psychiatry.

[31] Goodman R (1997). The strengths and difficulties questionnaire: a research note. J Child Psychol Psychiatry.

[32] Petersen AC, Crockett L, Richards M, Boxer A (1988). A self-report measure of pubertal status: reliability, validity, and initial norms. J Youth Adolesc.

[33] Petermann FP (2011) U. (Hrsg.) Wechsler Intelligence Scale for Children—Fourth Edition. Manual 1: Grundlagen, Testauswertung und Interpretation. Übersetzung und Adaptation der WISC-IV von David Wechsler. Frankfurt: Pearson

[34] Grosbras MH, Paus T (2006). Brain networks involved in viewing angry hands or faces. Cerebral Cortex (New York, NY: 1991)..

[35] Davids M, Zöllner FG, Ruttorf M, Nees F, Flor H, Schumann G (2014). Fully-automated quality assurance in multi-center studies using MRI phantom measurements. Magn Reson Imaging.

[36] Allen EA, Erhardt EB, Calhoun VD (2012). Data visualization in the neurosciences: overcoming the curse of dimensionality. Neuron.

[37] Zandbelt B (2017) Slice Display. figshare

[38] Truedsson E, Fawcett C, Wesevich V, Gredeback G, Wahlstedt C (2019). The role of callous-unemotional traits on adolescent positive and negative emotional reactivity: a longitudinal community-based study. Front Psychol.

[39] Andrade BF, Tannock R (2013). The direct effects of inattention and hyperactivity/impulsivity on peer problems and mediating roles of prosocial and conduct problem behaviors in a community sample of children. J Atten Disord.

[40] Smaragdi A, Cornwell H, Toschi N, Riccelli R, Gonzalez-Madruga K, Wells A (2017). Sex differences in the relationship between conduct disorder and cortical structure in adolescents. J Am Acad Child Adolesc Psychiatry.

[41] Prendergast BJ, Zucker I (2018). Social behavior: developmental timing defies puberty. Curr Biol: CB.

[42] Girard L-C, Tremblay RE, Nagin D, Côté SM (2019). Development of aggression subtypes from childhood to adolescence: a group-based multi-trajectory modelling perspective. J Abnorm Child Psychol.

[43] Coccaro EF, McCloskey MS, Fitzgerald DA, Phan KL (2007). Amygdala and orbitofrontal reactivity to social threat in individuals with impulsive aggression. Biol Psychiat.

[44] Besteher B, Squarcina L, Spalthoff R, Bellani M, Gaser C, Brambilla P (2017). Brain structural correlates of irritability: findings in a large healthy cohort. Hum Brain Mapp.

[45] Holz NE, Boecker R, Hohm E, Zohsel K, Buchmann AF, Blomeyer D (2015). The long-term impact of early life poverty on orbitofrontal cortex volume in adulthood: results from a prospective study over 25 years. Neuropsychopharmacol: Off Publ Am Coll Neuropsychopharmacol.

[46] Murray AL, Eisner M, Obsuth I, Ribeaud D (2017). Situating violent ideations within the landscape of mental health: associations between violent ideations and dimensions of mental health. Psychiatry Res.

[47] Wertz J, Agnew-Blais J, Caspi A, Danese A, Fisher HL, Goldman-Mellor S (2018). From childhood conduct problems to poor functioning at age 18 years: examining explanations in a longitudinal cohort study. J Am Acad Child Adolesc Psychiatry.

[48] Leadbeater BJ, Homel J (2015). Irritable and defiant sub-dimensions of ODD: their stability and prediction of internalizing symptoms and conduct problems from adolescence to young adulthood. J Abnorm Child Psychol.

[49] Wakschlag LS, Perlman SB, Blair RJ, Leibenluft E, Briggs-Gowan MJ, Pine DS (2018). The neurodevelopmental basis of early childhood disruptive behavior: irritable and callous phenotypes as exemplars. Am J Psychiatry.

[50] Brown BB (2004) Adolescents’ relationships with peers. Handbook of adolescent psychology. 363–394

[51] Steinberg L, Monahan KC (2007). Age differences in resistance to peer influence. Dev Psychol.

[52] Eagly AH, Wood W (2013). The nature-nurture debates: 25 years of challenges in understanding the psychology of gender. Perspect Psychol Sci: J Assoc Psychol Sci.

[53] Bartels M, Cacioppo JT, van Beijsterveldt TC, Boomsma DI (2013). Exploring the association between well-being and psychopathology in adolescents. Behav Genet.

[54] Kendler KS, Gardner CO (2014). Sex differences in the pathways to major depression: a study of opposite-sex twin pairs. Am J Psychiatry.

[55] Nyborg H (2005). Sex-related differences in general intelligence g, brain size, and social status. Personality Individ Differ.

