# Supplementary material for: Orbitofrontal control of conduct problems? Evidence from healthy adolescents processing negative facial affect
Source: Eur Child Adolesc Psychiatry. 2021 Apr 16;31(8):1–10. doi: 10.1007/s00787-021-01770-1 (PMC9343289; doi:10.1007/s00787-021-01770-1)
Supplement: Supplementary file 1 — Supplementary file1 (DOCX 139 KB) [file 787_2021_1770_MOESM1_ESM.docx]

**Supplemental information to manuscript: Orbitofrontal Control of Conduct Problems?**

**Evidence from healthy Adolescents Processing Negative Facial Affect**


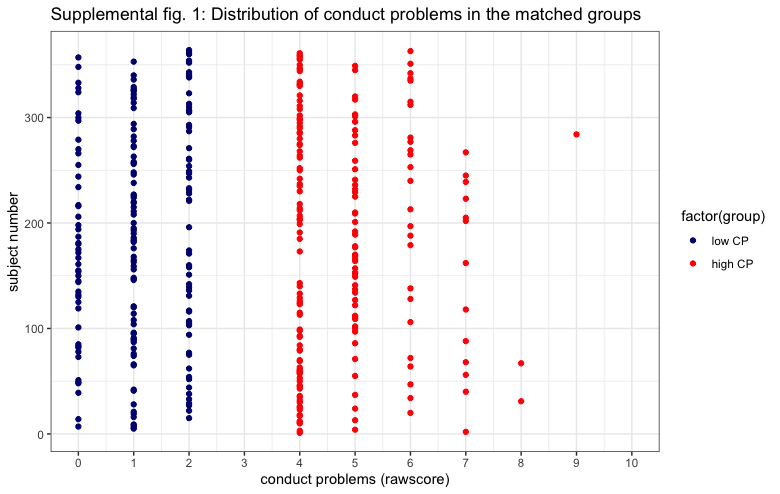


**Supplemental table A: Task effect of the faces task within matched subsample (N=364)**

| *Table A: Task effect of the faces task within matched subsample (n=364, FWE-corrected, k=0)* | | | | |
| --- | --- | --- | --- | --- |
| **Hemisphere** | **Label** | **MNI (x.y.z)** | **Cluster size** | **T-value** |
| L | anterior cingulate cortex | 0, 50, 7 | 966 | 9.73 |
| L | middle cingulate gyrus | 0, -25, 37 | 208 | 7.58 |
| L | posterior cingulate gyrus | -6, -52, 10 | 186 | 6.53 |
| R | medial frontal gyrus | 27, 47, 34 | 26 | 6.48 |
| R | fusiform gyrus | 27, -67, -11 | 18 | 6.26 |
| L | cuneus | -9, -97, 16 | 19 | 6.23 |
| R | lingual gyrus | 30, -34, -8 | 14 | 6.13 |
| L | lingual gyrus | -12, -91, -5 | 15 | 5.94 |
| R | lingual gyrus | 3, -79, -8 | 9 | 5.68 |
| L | fusiform gyrus | -30, -61, -14 | 12 | 5.60 |
| R | angular gyrus | 51, -58, 40 | 8 | 5.57 |
| L | anterior insula | -33, 14, -14 | 18 | 5.47 |

**Supplemental tables B1 & B2: Tests of differences in demographic and behavioral variables between sites (B1: high CP group, B2: low CP group)**

| *Table B1: ANOVA pairwise comparisons between sites in the low CP group* | | | | | | | | |
| --- | --- | --- | --- | --- | --- | --- | --- | --- |
| high CP | site1 (18) | site 2 (24) | site 3 (7) | site 4 (16) | site 5 (36) | site6 (27) | site7 (26) | site8 (28) |
| site 1 | - | - | - | - | - | - | - | Age,p=.031 |
| site 2 |  | - | - | - | PB,p=.042 | - | - | - |
| site 3 |  |  | - | - | - | - | - | - |
| site 4 |  |  |  | - | - | - | - | - |
| site 5 |  |  |  |  | - | - | - | - |
| site 6 |  |  |  |  |  | - | - | - |
| site 7 |  |  |  |  |  |  | - | - |
| site 8 |  |  |  |  |  |  |  | - |
| PB=prosocial behavior, brakets=number of subjects, p=value of the significant pairwise comparison | | | | | | | | |

| *Table B2: ANOVA pairwise comparisons between sites in the high CP group* | | | | | | | | |
| --- | --- | --- | --- | --- | --- | --- | --- | --- |
| low CP | site1 (29) | site2 (31) | site 3 (14) | site 4 (16) | site 5 (28) | site6 (24) | site7 (18) | site8 (22) |
| site 1 | - | - | - | - | - | - | - | - |
| site 2 |  | - | - | - | - | - | - | - |
| site 3 |  |  | - | - | - | - | - | IQ,p=.004 |
| site 4 |  |  |  | - | - | - | - | - |
| site 5 |  |  |  |  | - | - | - | - |
| site 6 |  |  |  |  |  | - | - | - |
| site 7 |  |  |  |  |  |  | - | - |
| site 8 |  |  |  |  |  |  |  | - |
| PB=prosocial behavior, brakets=number of subjects, p=value of the significant pairwise comparison | | | | | | | | |

**Dimensional analyses across full sample (N=1444)**

**Supplemental tables C1 & C2 (multiple regression on amygdala in the full sample):**

| *C1: Left amygdala in the full-sample (N=1444, angry >neutral) [model not sig.]* | | | | | |
| --- | --- | --- | --- | --- | --- |
| **Variables** | **B** | **Std. Error** | **Beta** | **t** | **Sig.** |
| (Constant) | .288 | .476 |  | .605 | .545 |
| SDQ_cond | .014 | .009 | .044 | 1.543 | .123 |
| SDQ_pros | .014 | .008 | .052 | 1.824 | .068 |
| Sex | -.053 | .026 | -.055 | -2.058 | .040 |
| Age | -.037 | .033 | -.031 | -1.135 | .257 |
| Pubertal Dev. | .001 | .009 | .004 | .148 | .883 |
| IQ | .002 | .001 | .045 | 1.690 | .091 |
| site | .004 | .005 | .021 | .794 | .427 |

| *C2: Right amygdala in the full sample (N=1444, angry >neutral) [model sig. F(7,1436)=3.91, p<.001]* | | | | | |
| --- | --- | --- | --- | --- | --- |
| **Variables** | **B** | **Std. Error** | **Beta** | **t** | **Sig.** |
| (Constant) | .555 | 417 |  | 1.332 | .183 |
| SDQ_cond | .005 | .008 | .019 | .667 | .505 |
| SDQ_pros | .022 | .007 | .089 | 3.133 | .002 |
| Sex | -.080 | .023 | -.094 | -3.556 | .000 |
| Age | -.055 | .028 | -.053 | -1.946 | .052 |
| Pubertal Dev. | .001 | .008 | .004 | .135 | .893 |
| IQ | .001 | .001 | .046 | 1.713 | .087 |
| site | .001 | .005 | .007 | .259 | .795 |

**Supplemental tables D1 – D3 (Multiple regression on OFC and ACC in the full sample):**

*D1: Right orbitofrontal cortex in the full sample (N=1444, angry >neutral) [model not sig.]*

| **Variables** | **B** | **Std. Error** | **Beta** | **t** | **Sig.** |
| --- | --- | --- | --- | --- | --- |
| (Constant) | -.104 | .263 |  | -.398 | .691 |
| Conduct Prob. | .007 | .005 | .040 | 1.389 | .165 |
| Prosocial Behav. | .006 | .004 | .040 | 1.401 | .161 |
| Sex | .001 | .014 | .003 | .098 | .922 |
| Age | .005 | .018 | .008 | .291 | .771 |
| Pubertal Dev. | .005 | .005 | .026 | .949 | .343 |
| IQ | -.001 | .001 | -.034 | -1.259 | .208 |
| site | .003 | .003 | .024 | .887 | .375 |

| *D2: Left anterior cingulate cortex in the full sample (N=1444, angry >neutral) [model not sig.]* | | | | | |
| --- | --- | --- | --- | --- | --- |
| **Variables** | **B** | **Std. Error** | **Beta** | **t** | **Sig.** |
| (Constant) | -.257 | .313 |  | -.823 | .410 |
| SDQ_cond | .003 | .006 | .014 | .485 | .627 |
| SDQ_pros | .008 | .005 | .046 | 1.591 | .112 |
| sex | .018 | .017 | .029 | 1.076 | .282 |
| Age | .015 | .021 | .019 | .698 | .485 |
| Pubertal Dev. | -.001 | .006 | -.006 | -.202 | .840 |
| IQ | .001 | .001 | .026 | .986 | .324 |
| site | -.002 | .004 | -.012 | -.439 | .661 |

| *D3: Right anterior cingulate cortex in the full sample (N=1444, angry >neutral) [model not sig.]* | | | | | |
| --- | --- | --- | --- | --- | --- |
| **Variables** | **B** | **Std. Error** | **Beta** | **t** | **Sig.** |
| (Constant) | -.150 | .305 |  | -.492 | .623 |
| SDQ_cond | .003 | .006 | .015 | .530 | .596 |
| SDQ_pros | .011 | .005 | .061 | 2.129 | .033 |
| sex | .022 | .017 | .036 | 1.357 | .175 |
| Age | .008 | .021 | .010 | .377 | .706 |
| Pubertal Dev. | -.002 | .006 | -.008 | -.292 | .771 |
| IQ | .000 | .001 | .018 | .654 | .513 |
| site | -.004 | .004 | -.034 | -1.268 | .205 |

**Supplemental table E1 (multiple regression on left OFC in the high CP group):**

| *E1: Left OFC in the high CP group (N=182, angry >neutral) [model sig. F(7,175)=3.08, p=.004]* | | | | | |
| --- | --- | --- | --- | --- | --- |
| **Variables** | **B** | **Std. Error** | **Beta** | **t** | **Sig.** |
| (Constant) | -.845 | .671 |  | 1.260 | .209 |
| Conduct Prob. | .057 | .017 | .253 | 3.406 | .001 |
| Prosocial Behav. | .021 | .009 | .183 | 2.406 | .017 |
| Sex | .031 | .035 | .066 | .898 | .370 |
| Age | .042 | .046 | .073 | .918 | .360 |
| Pubertal Dev. | -.014 | .013 | -.082 | -1.05 | .293 |
| IQ | -.001 | .001 | -.059 | -.803 | .423 |
| site | .013 | .008 | .127 | 1.678 | .095 |

**Supplemental table F1 & F2: supplemental analysis of left OFC without prosocial behavior /without CP in the full sample (multiple regressions)**

| *F1: Left OFC without prosocial behavior in the full sample (N=1444, angry >neutral) [model not sig.]* | | | | | |
| --- | --- | --- | --- | --- | --- |
| **Variables** | **B** | **Std. Error** | **Beta** | **t** | **Sig.** |
| (Constant) | -.230 | .264 |  | -.871 | .384 |
| Conduct Prob. | .006 | .005 | .033 | 1.228 | .220 |
| Sex | .020 | .014 | .036 | 1.367 | .172 |
| Age | .013 | .018 | .019 | .689 | .491 |
| Pubertal Dev. | .009 | .005 | .048 | 1.751 | .080 |
| IQ | -.001 | .001 | -.027 | -1.000 | .318 |
| site | .002 | .003 | .017 | .648 | .517 |

**Dimensional effect of prosocial behavior on left OFC**

In the full sample we also found a significant positive linear relationship between prosocial behavior and left OFC activity (ß=.066, p=.021, see table 2). Re-conducting the regression analysis with prosocial behavior as a covariate of interest (leaving CP out of the equation) yielded no significant effect of prosocial behavior on left OFC activity (see supplemental table F2).

| *F2: Right OFC without prosocial behavior in the full sample (N=1444, angry >neutral) [model not sig.]* | | | | | |
| --- | --- | --- | --- | --- | --- |
| **Variables** | **B** | **Std. Error** | **Beta** | **t** | **Sig.** |
| (Constant) | -.280 | .266 |  | -1.053 | .293 |
| Prosocial Behav. | .007 | .004 | .044 | 1.669 | .095 |
| Sex | .016 | .014 | .030 | 1.107 | .268 |
| Age | .013 | .018 | .020 | .725 | .469 |
| Pubertal Dev. | .009 | .005 | .048 | 1.742 | .082 |
| IQ | -.001 | .001 | -.027 | -1.001 | .317 |
| site | .003 | .004 | .044 | 1.669 | .095 |

**Dimensional analyses across matched groups (n=364)**

**Supplemental tables G1 & G2 (Multiple regression on amygdala across groups):**

| *G1:* *Left amygdala across groups (N=364, angry >neutral) [model sig. F(7.356)=3.72. p=.001]* | | | | | |
| --- | --- | --- | --- | --- | --- |
| **Variables** | **B** | **Std. Error** | **Beta** | **t** | **Sig.** |
| (Constant) | 4.437 | 1.379 |  | 3.218 | .001 |
| Conduct Prob. | -.013 | .020 | -.041 | -.690 | .491 |
| Prosocial Behav. | .005 | .021 | .015 | .252 | .801 |
| Sex | .027 | .074 | .019 | .370 | .712 |
| Age | -.306 | .094 | -.180 | -3.246 | .001 |
| Pubertal Dev. | -.014 | .028 | -.027 | -.486 | .627 |
| IQ | -.001 | .003 | -.024 | -.451 | .652 |
| site | .056 | .015 | .199 | 3.789 | .000 |

| *G2:* *Right amygdala across groups (N=364, angry >neutral) [model not sig.]* | | | | | |
| --- | --- | --- | --- | --- | --- |
| **Variables** | **B** | **Std. Error** | **Beta** | **t** | **Sig.** |
| (Constant) | 1.858 | .872 |  | 2.131 | .034 |
| Conduct Prob. | .016 | .012 | .079 | 1.323 | .187 |
| Prosocial Behav. | .026 | .013 | .119 | 1.954 | .051 |
| Sex | -0.050 | .047 | -.058 | -1.077 | .282 |
| Age | -0.148 | .060 | -.140 | -2.473 | .014 |
| Pubertal Dev. | .008 | .018 | .024 | .429 | .668 |
| IQ | .000 | .002 | -.013 | -.245 | .807 |
| site | .006 | .009 | .034 | .627 | .531 |

**Supplemental tables H1-H4 (multiple regression on OFC and ACC across groups):**

| *H1: Left orbitofrontal cortex across groups ( N=364, angry >neutral) model sig.[F(7,356=2.53), p=.015]* | | | | | | |
| --- | --- | --- | --- | --- | --- | --- |
| **Variables** | | **B Std. Error** | | **Beta** | **t** | **Sig.** |
|  | (Constant) | -.416 | .471 |  | -.882 | .378 |
|  | Conduct Prob. | .023 | .007 | .210 | 3.516 | .000 |
|  | Prosocial Behav. | .013 | .007 | .113 | 1.869 | .062 |
|  | Sex | .039 | .025 | .083 | 1.547 | .123 |
|  | Age | .008 | .032 | .014 | .254 | .799 |
|  | PDS | .005 | .009 | .032 | .576 | .565 |
|  | IQ | .001 | .001 | .035 | .656 | .512 |
|  | Site | .004 | .005 | .044 | .822 | .412 |
|  |  | | | | | |

*H2: Right orbitofrontal cortex across groups (N=364, angry >neutral) [model not sig.]*

| **Variables** | **B** | **Std. Error** | **Beta** | **t** | **Sig.** |
| --- | --- | --- | --- | --- | --- |
| (Constant) | -.213 | .481 |  | -.442 | .659 |
| Conduct Prob. | .005 | .007 | .049 | .798 | .425 |
| Prosocial Behav. | .007 | .007 | .058 | .932 | .352 |
| Sex | -.006 | .025 | -.014 | -.249 | .804 |
| Age | .007 | .033 | .013 | .226 | .821 |
| Pubertal Dev. | .003 | .010 | .018 | .314 | .754 |
| IQ | .000 | .001 | .010 | .184 | .854 |
| site | .002 | .005 | .023 | .414 | .679 |

H3: *Left anterior cingulate across groups (N=364, angry >neutral) [model not sig.]*

| **Variables** | **B** | **Std. Error** | **Beta** | **t** | **Sig.** |
| --- | --- | --- | --- | --- | --- |
| (Constant) | .783 | .555 |  | 1.410 | .159 |
| Conduct Prob. | .001 | .008 | .011 | .189 | .850 |
| Prosocial Behav. | .007 | .008 | .052 | .858 | .392 |
| Sex | .055 | .030 | .102 | 1.865 | .063 |
| Age | -.059 | .038 | -.089 | 1.556 | .120 |
| Pubertal Dev. | .003 | .011 | .015 | .258 | .797 |
| IQ | .001 | .001 | .034 | .620 | .535 |
| site | .005 | .006 | .042 | .772 | .441 |

*H4: Right anterior cingulate across groups (N=364, angry >neutral) [model not sig.]*

| **Variables** | **B** | **Std. Error** | **Beta** | **t** | **Sig.** |
| --- | --- | --- | --- | --- | --- |
| (Constant) | .488 | .557 |  | .875 | .382 |
| Conduct Prob. | .006 | .008 | .049 | .811 | .418 |
| Prosocial Behav. | .012 | .008 | .088 | 1.460 | .145 |
| Sex | .060 | .030 | .110 | 2.018 | .044 |
| Age | -.040 | .038 | -.061 | 1.060 | .290 |
| Pubertal Dev. | -.005 | .011 | -.024 | -.419 | .675 |
| IQ | .001 | .001 | .054 | .985 | .325 |
| site | .001 | .006 | .007 | .137 | .891 |
